# Racial and Ethnic Disparities in Preventive and Chronic Disease Care in Medicare Advantage vs. Traditional Medicare

**DOI:** 10.1007/s11606-025-09793-z

**Published:** 2025-08-11

**Authors:** Renuka Tipirneni, Andrei R. Stefanescu, Dominic A. Ruggiero, Alexandra G. Hames, John Z. Ayanian, Eric T. Roberts

**Affiliations:** 1 Division of General Medicine, Department of Internal Medicine, University of Michigan Medical School, Ann Arbor, MI, USA; 2 Institute for Healthcare Policy & Innovation, University of Michigan, Ann Arbor, MI, USA; 3 Division of General Internal Medicine, University of Pennsylvania Perelman School of Medicine, Philadelphia, PA, USA; 4 Department of Health Policy and Management, University of Pittsburgh Graduate School of Public Health, Pittsburgh, PA, USA; 5 Gerald R. Ford School of Public Policy, University of Michigan, Ann Arbor, MI, USA; 6 School of Public Health, University of Michigan, Ann Arbor, MI, USA; 7 Leonard Davis Institute of Health Economics, University of Pennsylvania, Philadelphia, USA

**Keywords:** medicare, chronic conditions, racial disparity

## Abstract

**BACKGROUND:**

Over half of Medicare beneficiaries are enrolled in Medicare Advantage (MA), with Black and Hispanic beneficiaries disproportionately in MA versus traditional Medicare (TM).

**OBJECTIVE:**

To examine Black-White and Hispanic-White disparities in preventive and chronic disease care by MA vs. TM.

**DESIGN:**

Cross-sectional propensity-score-weighted difference-in-disparities analyses compared Black-White and Hispanic-White disparities in MA and TM using the Medicare Current Beneficiary Survey (2015–2020).

**PARTICIPANTS:**

Medicare beneficiaries with cardiovascular disease or risk factors (N = 68,788 person-years).

**MAIN MEASURES:**

Influenza vaccine, pneumococcal vaccine, blood pressure check, cholesterol test, colorectal cancer screening, preventive care index (count of above; 0–5), mammogram, annual wellness visit; hemoglobin A1C and eye exam.

**KEY RESULTS:**

Black and Hispanic, compared to white, beneficiaries were less likely to receive annual wellness visits, influenza vaccines, pneumococcal vaccines, and colorectal cancer screening. Black beneficiaries in MA vs. TM had higher overall preventive care use (preventive care index, 3.67 vs. 3.44) and higher rates of all preventive services examined. Hispanic beneficiaries in MA vs. TM had higher preventive care use (index, 3.67 vs. 3.56), including annual wellness visit, blood pressure check, colorectal cancer screening, and breast cancer screening. Preventive care use was higher among White beneficiaries in MA than TM (index, 3.88 and 3.79). Black-White disparities were smaller in MA than TM for preventive care use (difference-in-disparities: + 0.13 index points, 95% CI 0.04–0.22), blood pressure check (+ 2.2 percentage points [p.p.], 95% CI 0.1–4.4), cholesterol check (+ 2.2 p.p., 95% CI 0.2–4.2), and eye exam (+ 5.0 p.p., 95% CI 1.4–8.7). Hispanic-White disparities were not statistically different in MA vs. TM.

**CONCLUSIONS:**

Although MA was associated with smaller Black-White disparities in preventive care compared to TM, these differences were modest, and MA was not associated with smaller Hispanic-White disparities.

## BACKGROUND

Cardiovascular disease is the leading cause of death in the US,^1^ and Black and Hispanic individuals, compared with non-Hispanic White individuals, face disproportionate burdens of cardiovascular disease and cardiovascular risk factors including hypertension, diabetes, and hyperlipidemia.^2,3^ However, Black and Hispanic older adults are less likely than non-Hispanic White older adults to see a doctor or take medicine to control chronic conditions such as diabetes.^4^ Access to high-quality chronic disease management and preventive care may ameliorate disparities in care and health outcomes.

Racial and ethnic disparities in chronic disease care are pervasive among older adults with Medicare^5–7^ and may differ according to the system in which Medicare coverage is delivered.^8–12^ For the first time in Medicare’s history, more than half of Medicare beneficiaries are enrolled in Medicare Advantage (MA), in which private health insurers deliver Medicare benefits, rather than the traditional Medicare (TM) program, which is federally administered.^13^ Furthermore, Black and Hispanic beneficiaries are disproportionately enrolled in MA.^14^ Yet little is known about whether disparities in Black and Hispanic vs. White beneficiaries’ use of preventive services and chronic disease care differ in MA vs. TM. MA plans frequently emphasize care management, have financial incentives linked to quality indicators including chronic disease care,^15^ and offer reduced cost-sharing for beneficiaries.^16^ These features of MA may encourage greater use of chronic disease care and preventive services, particularly in marginalized populations.^17,18^ Conversely, TM has broader provider networks than MA and fewer restrictions on utilization, unlike MA plans that may employ narrow clinician networks and utilization management (e.g., prior authorization)^19^ to restrict higher-cost services.

Although several studies have demonstrated an association of MA with smaller racial and ethnic disparities,^20–24^ others found few differences in disparities compared to TM.^25^ A recent systematic review concluded there was insufficient evidence on whether MA mitigates racial and ethnic disparities relative to TM.^26^ In addition, most of these studies used data from periods before MA became the predominant mode of coverage in the Medicare program. Therefore, using national survey data for Medicare beneficiaries, we examined racial and ethnic differences in preventive services and chronic disease care by MA versus TM among beneficiaries with cardiometabolic disease and assessed whether MA was associated with smaller racial and ethnic disparities in care.

## METHODS

### Study Design

We used Medicare Current Beneficiary Survey (MCBS) data from 2015–2020. The MCBS is a longitudinal survey that collects data on a nationally representative sample of Medicare beneficiaries across several domains, including health care utilization and costs, experiences with care, and health status.^27^ Participants are interviewed in-person or by phone up to eleven times over four years. During the COVID-19 pandemic, data collection was converted to phone-only.

Respondents were included in our analysis if they reported that a doctor had ever diagnosed them with at least one of the following cardiometabolic diseases or related risk factors: myocardial infarction, angina, atherosclerosis, stroke, congestive heart failure, other heart conditions, diabetes, hypertension, or hyperlipidemia. Respondents were excluded if they lived in an institutional setting rather than in the community or outside the 50 US states and District of Columbia. Our final sample size was N = 72,054 person-years (Appendix Fig. 1).

We also conducted two sets of analyses in subsamples of MCBS respondents. First, we examined diabetes care indicators among individuals who reported a diagnosis of diabetes (N = 26,724 person-years). Second, we analyzed the subset of respondents who participated in the MCBS Cost Supplement, which includes health care use reported by MA and TM beneficiaries and from linked Medicare claims. We used Cost Supplement data for secondary analyses of medication adherence to statins, antihypertensive drugs, and diabetes drugs. The samples for these analyses were N = 16,579, 22,402, and 6,549 person-years, respectively.

### Variables

We used two main predictors. First, Medicare coverage was dichotomized as MA or TM (see Appendix Methods). Second, we used respondent-reported race and ethnicity to categorize respondents as Black, Hispanic, or non-Hispanic White. We did not classify Black race and Hispanic ethnicity as mutually exclusive to account for individuals who identified as Black and Hispanic. This specification is consistent with the recommendations of the US Census Bureau.^28^

Our primary outcomes were measured annually based on self-report and included annual wellness visit since last fall (available in 2020 only), blood pressure check (past six months), blood cholesterol check (past year), influenza vaccine (past year), pneumococcal vaccine (ever), colorectal cancer screening (ever), breast cancer screening (past year, among women), hemoglobin A1C check (2 or more times in past year, among respondents with diabetes), and eye exam in past year (among respondents with diabetes). We also defined a preventive care index as the sum of the indicators for blood pressure check, cholesterol check, influenza vaccine, pneumococcal vaccine, and colorectal cancer screening (0–5 scale for the number of “yes” responses). We included these services in the index because they reflect key aspects of preventive care, applied to all participants regardless of sex or diabetes diagnosis, and were available in all study years.

Covariates included age (years), sex, highest education level, marital status, smoking history (yes/no), activities of daily living score, metropolitan residence status (metropolitan, micropolitan, or rural), reason for Medicare eligibility (age, disability, or end-stage renal disease), and a chronic disease comorbidity index. The activities of daily living score was the sum of indicators for limitations to either activities of daily living or instrumental activities of daily living: difficulty bathing/showering, dressing, eating, getting in or out of bed/chair, walking, using the toilet, using the telephone, doing light housework, doing heavy housework, preparing meals, shopping, and managing money (0–12 scale, depending on number of “yes” responses). The chronic disease comorbidity index was a count of the following self-reported conditions: diabetes, atherosclerosis, hypertension, myocardial infarction, stroke, heart failure, angina, hyperlipidemia, other heart condition, skin cancer, cancer other than skin cancer, depression, arthritis, Alzheimer’s disease, dementia, osteoporosis, hearing loss, and vision loss. Except for Medicare eligibility, all covariates were self-reported by respondents.

### Statistical Analysis

All analyses were weighted to account for the MCBS complex sample design. We used propensity score weighting to statistically balance the probability of our race and ethnicity groups having MA versus TM coverage, using sociodemographic and health characteristics including age, sex, education level, marital status, smoking history, activities of daily living score, metropolitan residence, reason for Medicare eligibility, and the chronic disease comorbidity index. Propensity score weights were developed using three separate weighted logistic regression models (one for each race/ethnicity group) with type of Medicare as the outcome and the demographics above as predictors. Final analytic weights were obtained by multiplying the MCBS survey weights with these propensity score weights.^29^

In each racial and ethnic group, we compared sociodemographic and health characteristics of beneficiaries before and after propensity score weighting and tested for differences between MA and TM enrollees using Rao-Scott Chi-Square tests (categorical variables) and linear regression (continuous variables). For our primary analysis assessing the difference-in-disparities, weighted logistic regression models were used to estimate interactions between type of Medicare coverage (i.e., MA vs. TM) and either race or ethnicity, adjusted for covariates. Disparities within MA, disparities within TM, and difference-in-disparities estimates are reported as weighted percentages for categorical variables or weighted means for continuous variables. Separate models were used to estimate Black-White and Hispanic-White differences. To facilitate interpretation, estimates of binary outcomes are reported as marginal effects, or percentages.

We conducted supplemental analyses using the same analytic method to assess the secondary outcome of medication adherence. In addition, we repeated the main analyses in a sample restricted to individuals reporting stroke or myocardial infarction, as these conditions are most likely to be diagnosed in an emergency or inpatient setting and, thus, less susceptible to concerns for upcoding due to additional MA plan screenings. Lastly, we assessed if MA vs. TM differences in the preventive care index varied by whether beneficiaries had Medicaid supplemental insurance (Appendix Methods).^30,31^

Statistical significance was defined a priori as α = 0.05. Tables report both standard *p* values and *p* values generated from a Benjamini–Hochberg adjustment for multiple comparisons. Analyses were conducted using SAS 9.4 and Stata 16.1.

## RESULTS

### Participant Demographics

Our final sample included 44,106 person-years with TM and 27,948 person-years with MA. Table 1 reports sample characteristics before propensity score weighting. Among beneficiaries in MA, the mean age was 72.4 (SD 0.13) years and 56.3% were female; among those in TM, the mean age was 71.4 (SD 0.14) years and 52.5% were female. Beneficiaries in MA were more likely to identify as Black or Hispanic, have lower incomes (≤ 200% of the poverty level), have lower educational attainment, and live in metropolitan areas, compared to those in TM. MA enrollees were slightly more likely than those in TM to report a history of diabetes, hypertension, or heart attack, but slightly less likely to report other cardiovascular disease. Propensity score weighting improved the balance between MA and TM enrollees on sociodemographic and health variables within racial and ethnic groups (Appendix Tables 1 and 2).

### Black―White Disparities.

In both MA and TM, Black beneficiaries were less likely to have received an annual wellness visit, influenza vaccine, pneumococcal vaccine, and colorectal cancer screening, compared to non-Hispanic White beneficiaries (Table 2). However, Black beneficiaries in both MA and TM were more likely to have received a blood pressure check, cholesterol check, and breast cancer screening than non-Hispanic White beneficiaries. Black beneficiaries had higher overall use of preventive care in MA, with a preventive care index of 3.67 (73.4% of services comprising the index) compared to 3.44 in TM (68.8% of services), implying use of an additional 0.23 preventive services, or a 4.6 percentage point (p.p) higher share of the five preventive services included in the index, in MA than in TM. Black beneficiaries in MA vs. TM had higher rates of annual wellness visits (44.9% vs. 29.5%), blood pressure checks (92.3% vs. 89.7%), cholesterol tests (94.8% vs. 91.6%), influenza vaccination (66.6% vs. 61.5%), pneumococcal vaccination (72.6% vs. 70.4%), and colorectal cancer screening (70.0% vs. 64.8%), all p < 0.05. Black beneficiaries in MA vs. TM also were more likely to have received breast cancer screening (52.0% vs. 47.1%), a hemoglobin A1C test (69.5% vs. 67.0%), and a diabetic eye exam (56.0% vs. 52.3%). Non-Hispanic White beneficiaries also had modestly higher rates of preventive care use in MA vs. TM (e.g., preventive care index, 3.88 in MA vs. 3.79 in TM), for an additional 0.09 of a preventive service or a 1.8 p.p. difference in the share of preventive services used.

Black-White disparities were smaller in MA than TM for the preventive care index (MA, −0.21 index points vs. TM, −0.35 index points). The corresponding difference-in-disparities estimate (+ 0.13 index points, 95% CI 0.04–0.22) indicates that MA was associated with a 2.6 p.p. reduction in the Black-White disparity in the share of preventive services used. Black-White disparities were smaller in MA vs. TM for blood pressure check (MA + 2.9 p.p. vs. TM + 0.7 p.p; difference-in-disparities + 2.2 p.p., 95% CI 0.1–4.4), cholesterol check (MA + 3.0 p.p vs. TM + 0.8 p.p; difference-in-disparities + 2.2 p.p., 95% CI 0.2–4.2), and eye exam (MA −0.6 p.p vs. TM −5.6 p.p; difference-in-disparities + 5.0 p.p., 95% CI 1.4–8.7). However, within MA, Black beneficiaries received fewer preventive services (including lower vaccination rates) than non-Hispanic White beneficiaries. There was no statistically significant difference in Black-White disparities between MA and TM for other individual services examined.

### Hispanic―White Disparities.

Compared to non-Hispanic White beneficiaries, Hispanic beneficiaries in both MA and TM had lower rates of annual wellness visits, influenza vaccine, pneumococcal vaccine, colorectal cancer screening, and hemoglobin A1C tests (Table 3). Hispanic beneficiaries in MA vs. TM had higher overall preventive care use (index 3.67 vs. 3.56), including higher rates of annual wellness visits (47.4% vs. 39.5%), blood pressure checks (90.4% vs. 88.3%), cholesterol tests (93.3% vs. 91.9%), colorectal cancer screening (71.4% vs. 66.0%), breast cancer screening (49.3% vs. 44.4%), and diabetic eye exams (59.1% vs. 57.8%). However, Hispanic individuals in MA vs. TM had lower rates of influenza vaccine (67.8% vs. 69.5%), pneumococcal vaccine (72.3% vs. 73.6%), and hemoglobin A1C test (59.7% vs. 61.6%), and non-Hispanic White beneficiaries had a higher overall rate of preventive service use in MA vs. TM (all p < 0.05).

Hispanic-White disparities were not statistically different in MA vs. TM for preventive or chronic disease services. For example, within TM, Hispanic beneficiaries received fewer preventive care services than non-Hispanic White beneficiaries (disparity in preventive care index: −0.23 index points, 95% CI: −0.30 to −0.16). Within MA, the Hispanic-White disparity in the preventive care index was −0.21 index points (95% CI: −0.27, 0.15).

### Supplemental Analyses.

In analyses of medication adherence, Black-White disparities were smaller for statin adherence in MA compared to TM (MA, −4.1 p.p. vs. TM, −8.9 p.p.; difference-in-disparities + 4.8 p.p., 95% CI 1.5–8.1) and Hispanic-White disparities were smaller for thiazolidinediones (MA, +1.0 p.p. vs. TM, +10.3 p.p.; difference-in-disparities + 11.3 p.p., 95% CI −0.2–22.8), but there were no significant differences in disparities across all other medication classes (Appendix Table 3).

Among beneficiaries with and without dual Medicaid enrollment, both Black and White beneficiaries had higher overall preventive service use in MA vs. TM, but the difference-in-disparities was not statistically significant (Appendix Table 4). There was also no difference in Hispanic-White disparities. In the sub-sample of individuals reporting stroke or myocardial infarction, the findings were overall similar though fewer of the difference-in-disparities estimates were statistically significant, likely reflecting reduced power in the sub-sample (Appendix Table 5).

## DISCUSSION

This nationally representative study found that Black and Hispanic Medicare beneficiaries with cardiovascular disease or cardiovascular risk factors had higher rates of preventive service use in MA compared with TM, yet lower overall levels of preventive service use than non-Hispanic White beneficiaries in these programs. Black-White disparities in preventive service use were somewhat smaller in MA vs. TM, yet were prevalent in both programs, and Hispanic-White disparities did not differ significantly between MA and TM. Thus, while greater use of preventive and chronic disease care in MA modestly narrowed the extent of Black-White disparities, disparities in care persisted across both modes of delivery of the Medicare program.

This study adds to prior research that compared racial and ethnic disparities in care between MA and TM, while using recent data to examine disparities across important preventive care indicators. Prior research has been inconclusive about whether MA is associated with fewer racial and ethnic disparities in health care access and use compared to TM.^26,32^ For example, some studies found that MA compared to TM was associated with lessened racial and ethnic disparities in diabetes care management and breast cancer screenings.^20,21^ However, multiple studies found inequities in both sectors of the Medicare program, with little evidence that MA was consistently associated with lessened Black-White and Hispanic-White disparities in access to care,^23,24,31^ preventive service use,^22,23,33^ or potentially avoidable hospitalizations.^26,34^

Aligned with this mixed evidence, we found that MA was associated with smaller Black-White, but not Hispanic-White, disparities in preventive care use, and that substantial disparities existed across both MA and TM. Although Black-White disparities in preventive care use were often statistically smaller in MA compared to TM, the differences were modest in magnitude and may or may not be clinically meaningful. For example, estimates of disparities for the preventive care index suggest that MA was associated with only a 2.6 percentage point reduction in the Black-White disparity in preventive service use. We also did not find a difference in Hispanic-White disparities in preventive service use between MA vs. TM.

Some MA features, including care management, quality benchmarks, and cost-sharing reductions, might lessen disparities by promoting more uniform use of preventive services.^14,18,35,36^ However, this study’s finding of racial and ethnic disparities in both MA and TM suggests that structural factors, irrespective of Medicare coverage type, likely contribute to inequities. Such factors can include medical mistrust,^37^ disparate access to clinicians,^38^ inequities in financial resources to pay for care, and differences in access to high-quality plans.^14,39,40^ Thus, an urgent need remains for policies to address these structural determinants of inequities alongside initiatives to incentivize equitable care delivery in both MA and TM.

Although research has highlighted disparities in both MA and TM, some studies suggest that the extent of racial and ethnic disparities varies across MA plans.^40^ For example, Park et al. found that Black-White disparities in potentially preventable hospitalization rates were larger in MA plans with lower star ratings (those rated ≤ 3.5 stars in the MA star rating system).^41^ Gupta et al. found lower rates of the highest-quality MA plans (those rated ≥ 4.5 stars) and lower mean star ratings among MA plans in counties deemed more socially vulnerable,^42^ suggesting that differences in access to high-quality MA plans may mediate disparities. Several studies also found wide variation in health care disparities across MA plans, including disparities in MA plans deemed to have high quality by star ratings.^8,43^ We did not have a sufficiently large sample of beneficiaries at the plan level to examine whether specific features of MA plans were linked to narrower disparities. Additionally, because our supplemental medication adherence analyses were potentially underpowered, future work should expand upon this exploratory analysis to further examine disparities in medication use.

### Limitations

This study has several limitations. First, because enrollment in MA is voluntary, unmeasured differences between individuals who choose MA versus TM may lead to residual confounding. Second, because MA includes a heterogeneous pool of private MA plans, any analyses comparing MA and TM need to be interpreted in light of that heterogeneity. Further work examining the extent to which disparities vary across MA plans categorized according to benefit design (e.g., cost-sharing), provider networks, and performance on quality indicators, could elucidate specific plan features that may be linked to smaller disparities. Third, we analyzed individuals who reported having a diagnosis of cardiometabolic disease and adjusted for other respondent-reported comorbidities. Using self-reported diagnoses, rather than administrative data, avoids biases from coding differences between MA and TM. However, differences in care patterns (e.g., differences in screenings leading to differential rates of diagnosis) between the programs could contribute to differences in how respondents report their health. Findings from sensitivity analyses in a sub-sample of those reporting stroke or myocardial infarction were largely similar to the main analysis, suggesting low concern for differential rates of diagnosis in the present analysis. Fourth, medication adherence analyses were conducted on smaller cohorts, and data on annual wellness visits was only collected in 2020, limiting power to detect disparities in these outcomes.

### Policy Implications

Despite these limitations, the study has key policy implications for advancing racial and ethnic equity in Medicare. First, ongoing research is needed to assess MA vs. TM disparities across other dimensions of care and in other beneficiary subpopulations. Second, it remains important to identify specific areas where quality of care for racial and ethnic subgroups varies within the MA program. Currently, quality of care is evaluated using a star rating system, which summarizes performance across quality indicators at the MA contract level. However, contract-level ratings aggregate outcomes across different subpopulations of beneficiaries, and often, across multiple plans serving different geographic regions. To monitor the care received by different racial/ethnic subgroups, policymakers should consider monitoring disparities at the MA plan level using measures such as the Health Equity Summary Score or patient-reported measures of equitable care.^44^ Third, consideration should be given to minimum standards of access and quality of care in MA plans, as plan features such as narrow networks and prior authorization requirements could disproportionately affect access for minoritized populations. Thus, efforts to ameliorate disparities in the quality of care within MA should account for the level at which equity standards are considered and enforced.

## CONCLUSIONS

In both MA and TM, Black and Hispanic individuals with cardiovascular disease or related risk factors received fewer preventive and chronic disease management services than non-Hispanic White individuals. Black and Hispanic beneficiaries received more preventive and chronic disease services in MA than in TM. MA was associated with smaller Black-White disparities in care, however these differences were modest, and MA was not linked to smaller Hispanic-White disparities.

## Supplementary Material

Supplemental file

Supplementary Information The online version contains supplementary material available at https://doi.org/10.1007/s1160-025-09793-z.

## Figures and Tables

**Table 1:** Medicare Current Beneficiary Survey Participant Characteristics in Traditional Medicare and Medicare Advantage

	No. (%)*		
Variable	Traditional Medicare (n = 44,106 person-years)	Medicare Advantage (n = 27,948 person-years)	*p* Value

**Race**			< 0.001
American Indian or Alaska Native	590 (1.3)	372 (1.2)	
Asian	813 (2.8)	484 (2.6)	
Black	4,003 (9.3)	3,553 (13.6)	
Hawaiian or Pacific Islander	75 (0.2)	36 (0.1)	
White	36,366 (83.1)	21,729 (78.8)	
Other	180 (0.3)	109 (0.4)	
More than one race	1,240 (3.1)	843 (3.3)	
**Ethnicity**			< 0.001
Hispanic	3,249 (6.8)	4,182 (12.7)	
Non-Hispanic	40,629 (93.2)	23,628 (87.3)	
**Age, mean (SD), years**	71.4 (0.14)	72.4 (0.13)	< 0.001
**Sex**			< 0.001
Female	23,351 (52.5)	15,637 (56.3)	
Male	20,755 (47.5)	12,311 (43.7)	
**Medicare eligibility reason**			< 0.001
Age	36,863 (85.1)	24,338 (86.9)	
Disability	6,669 (13.8)	3,480 (12.7)	
End stage renal disease	574 (1.1)	130 (0.4)	
**Education**			< 0.001
Less than high school	7,140 (13.7)	6,181 (19.1)	
High school	12,238 (25.7)	7,931 (27.6)	
More than high school	24,544 (60.6)	13,722 (53.3)	
**Marital status**			< 0.001
Married	21,672 (54.5)	13,254 (51.0)	
Widowed	11,540 (20.8)	7,569 (22.1)	
Divorced or separated	6,448 (16.6)	4,814 (19.6)	
Never married	4,418 (8.2)	2,293 (7.3)	
**Area of residence**			< 0.001
Metropolitan	31,292 (76.6)	23,287 (86.4)	
Micropolitan	8,358 (15.4)	2,787 (8.1)	
Rural	4,450 (8.0)	1,872 (5.5)	
**ADL and IADL limitations,**† **mean (SD)**	1.56 (0.03)	1.56 (0.03)	0.90
**Responding to MCBS using a proxy**	4,623 (8.6)	2,585 (7.3)	< 0.001
**History of chronic illness**			
Diabetes	15,746 (36.7)	10,978 (40.3)	< 0.001
Hyperlipidemia	31,572 (73.0)	20,571 (73.7)	0.19
Hypertension	32,615 (73.1)	21,041 (74.4)	0.03
Atherosclerosis	4,295 (9.1)	2,506 (8.8)	0.43
Myocardial infarction	5,656 (11.9)	3,675 (12.8)	0.03
Angina	4,859 (10.4)	2,957 (10.5)	0.92
Congestive heart failure	3,984 (8.0)	2,354 (7.9)	0.68
Stroke	5,641 (11.6)	3,548 (11.9)	0.43
Other cardiovascular disease	14,281 (29.7)	7,785 (26.8)	< 0.001
Skin cancer	10,644 (21.8)	5,682 (18.8)	< 0.001
Other cancer	8,999 (19.2)	5,470 (18.7)	0.38
Arthritis	9,251 (19.8)	6,291 (21.5)	0.004
Osteoporosis	8,130 (17.0)	5,261 (18.3)	0.008
Alzheimer’s disease	1,078 (1.9)	748 (2.0)	0.48
Other dementia	1,449 (2.6)	1,084 (3.1)	0.007
Depression	12,307 (27.7)	7,789 (28.4)	0.36
Vision loss	3,736 (9.1)	2,461 (9.2)	0.96
Hearing loss	7,795 (18.1)	4,952 (17.6)	0.32
**Number of chronic illnesses, mean (SD)**	3.95 (0.03)	3.99 (0.03)	0.17

Abbreviations: ADL, activities of daily living; IADL, instrumental activities of daily living; MCBS, Medicare Current Beneficiary Survey

* Not propensity-score weighted

† The ADL and IADL limitations score is calculated by summing indicators for the following activities of daily living: difficulty bathing/showering, dressing, eating, getting in or out of bed/chair, walking, using the toilet, using the telephone, doing light housework, doing heavy housework, preparing meals, shopping, and managing money

**Table 2:** Preventive and Chronic Disease Care for Black and Non-Hispanic White Medicare Beneficiaries in Traditional Medicare and Medicare Advantage*

Measures	Black^†^ (n = 7,315)^‡^		Non-Hispanic White (n = 61,940)		Absolute difference, Black–Non-Hispanic White^§^		Difference in disparities (DID), MA vs. TM, mean or p.p. (95% CI)^§^	Unadjusted *p* values for DID	Benjamini–Hochberg Adjusted *p* values for DID
	MA %	TM %	MA %	TM %	MA, p.p (95% CI)	TM, p.p (95% CI)			

*Preventive Care*									
Preventive care index^‖^	3.67	3.44	3.88	3.79	**−0.21 (−0.27, −0.15)**	**−0.35 (−0.42, −0.27)**	**0.13 (0.04, 0.22)**	**0.004**	**0.04**
(0–5), mean Annual wellness	44.9	29.5	55.4	46.0	**−10.5 (−15.3, −5.8)**	**−16.6 (−22.0, −11.2)**	6.0 (−1.1, 13.2)	0.10	0.17
visit^¶^ Blood pressure check	92.3	89.7	89.4	89.1	**2.9 (1.6, 4.1)**	0.7 (−1.1, 2.4)	**2.2 (0.1, 4.4)**	**0.04**	0.10
Cholesterol test	94.8	91.6	91.8	90.8	**3.0 (1.9, 4.1)**	0.8 (−0.9, 2.5)	**2.2 (0.2, 4.2)**	**0.03**	0.11
Influenza vaccine	66.6	61.5	77.6	75.8	**−11.1 (−14.0, −8.1)**	**−14.3 (−17.5, −11.1)**	3.3 (−1.0, 7.5)	0.13	0.17
Pneumococcal vaccine	72.6	70.4	83.9	83.0	**−11.3 (−14.1, −8.5)**	**−12.6 (−15.7, −9.5)**	1.3 (−2.8, 5.4)	0.53	0.53
Colorectal cancer screening	70.0	64.8	72.6	69.8	**−2.6 (−4.5, −0.7)**	**−5.0 (−7.3, −2.7)**	2.4 (−0.5, 5.3)	0.11	0.15
Breast cancer screening	52.0	47.1	44.5	44.1	**7.6 (4.5, 10.6)**	3.1 (−0.7, 6.8)	4.5 (−0.3, 9.3)	0.07	0.13
*Diabetes Management* ^#^									
Hemoglobin A1C (≥ 2 in past year)	69.5	67.0	65.6	65.6	3.8 (−0.5, 8.1)	1.4 (−3.5, 6.4)	2.4 (−4.1, 8.9)	0.47	0.52
Eye exam	56.0	52.3	56.6	58.0	−0.6 (−3.1, 1.9)	**−5.6 (−8.3, −2.9)**	**5.0 (1.4, 8.7)**	**0.007**	**0.04**

Abbreviations: TM, Traditional Medicare; MA, Medicare Advantage; p.p., percentage points; DID, difference in disparities

* Results are propensity score weighted

† Black individuals were identified based on self-reported race; individuals who identified as both Black and Hispanic were included in both sets of analyses

‡ n values reported are in person-years

§ Bolded numbers indicates significance at *p* < 0.05 for unadjusted *p* values

‖ Preventive care index is a sum of indicators for blood pressure check, cholesterol test, influenza vaccine, pneumococcal vaccine, and colorectal cancer screening (range 0–5, depending on number of “yes” responses)

¶ Annual wellness visit data were collected only in 2020

# Sample is restricted to only participants who reported a diabetes diagnosis

**Table 3:** Preventive and Chronic Disease Care for Hispanic and Non-Hispanic White Medicare Beneficiaries in Traditional Medicare and Medicare Advantage*

Measures	Hispanic^†^ (n = 7,431)^‡^		Non-Hispanic White (n = 61,940)		Absolute difference, Hispanic–Non-Hispanic White^§^		Difference in disparities (DID), MA vs. TM, mean or p.p. (95% CI)^§^	Unadjusted *p* values for DID	Benjamini–Hochberg Adjusted *p* values for DID
	MA %	TM %	MA %	TM %	MA, p.p (95% CI)	TM, p.p (95% CI)			

*Preventive Care*									
Preventive care index^‖^	3.67	3.56	3.88	3.79	**−0.21 (−0.27, −0.15)**	**−0.23 (−0.30, −0.16)**	0.02 (−0.07, 0.11)	0.62	0.77
(0–5), mean Annual wellness visit^¶^	47.4	39.5	55.5	46.0	**−8.1 (−13.0, −3.2)**	**−6.5 (−12.5, −0.5)**	−1.6 (−9.3, 6.2)	0.69	0.77
Blood pressure check	90.4	88.3	89.4	89.1	1.0 (−0.4, 2.4)	−0.7 (−2.6, 1.1)	1.7 (−0.6, 4.0)	0.14	0.35
Cholesterol test	93.3	91.9	91.8	90.8	**1.4 (0.2, 2.7)**	**1.1 (0.2, 2.7)**	0.3 (−1.6, 2.3)	0.73	0.73
Influenza vaccine	67.8	69.5	77.6	75.8	**−9.9 (−12.7, −7.1)**	**−6.4 (−9.7, −3.0)**	−3.5 (−7.7, 0.7)	0.11	0.53
Pneumococcal vaccine	72.3	73.6	83.9	83.0	**−11.6 (−14.3, −8.8)**	**−9.5 (−12.7, −6.3)**	−2.1 (−6.2, 2.1)	0.32	0.54
Colorectal cancer screening	71.4	66.0	72.6	69.8	−1.3 (−3.2, 0.7)	**−3.8 (−6.2, −1.3)**	2.5 (−0.6, 5.6)	0.11	0.36
Breast cancer screening	49.3	44.4	44.5	44.1	**4.8 (1.7, 7.9)**	0.4 (−3.7, 4.4)	4.4 (−0.6, 9.5)	0.08	0.83
Diabetes Management^#^ Hemoglobin A1C (≥ 2 in past year)	59.7	61.6	65.6	65.6	**−6.0 (−10.3, −1.7)**	−4.0 (−9.3, 1.4)	−2.0 (−8.9, 4.8)	0.56	0.80
Eye exam	59.1	57.8	56.6	58.0	**2.5 (0.1, 4.9)**	−0.2 (−3.0, 2.7)	2.6 (−1.1, 6.3)	0.16	0.32

Abbreviations: TM, Traditional Medicare; MA, Medicare Advantage; p.p., percentage points; DID, difference in disparities

* Results are propensity score weighted

† Hispanic individuals were identified based on self-reported ethnicity; individuals who identified as both Black and Hispanic were included in both sets of analyses

‡ n values reported are in person-years

§ Bolded numbers indicates significance at *p* < 0.05 for unadjusted *p* values

‖ Preventive care index is a sum of indicators for blood pressure check, cholesterol test, influenza vaccine, pneumococcal vaccine, and colorectal cancer screening (range 0–5, depending on number of “yes” responses)

¶ Annual wellness visit data were collected only in 2020

# Sample is restricted to only participants who reported a diabetes diagnosis

## Data Availability

The data for this study were made available to the study team through a Data Use Agreement with the Centers for Medicare and Medicaid Services. Previously presented: Racial and Ethnic Disparities in Preventive and Chronic Disease Care in Medicare Advantage vs. Traditional Medicare. Poster presentation, Society of General Internal Medicine Annual Meeting, Boston, MA, May 2024; Racial and Ethnic Disparities in Preventive and Chronic Disease Care in Medicare Advantage vs. Traditional Medicare. Poster presentation, AcademyHealth Annual Research Meeting, Baltimore, MD, June-July 2024.
