# Supplementary material for: Racial and Ethnic Disparities in Preventive and Chronic Disease Care in Medicare Advantage vs. Traditional Medicare
Source: J Gen Intern Med. Author manuscript; Available in PMC 2026 Feb 19. (PMC12914165; doi:10.1007/s11606-025-09793-z)
Supplement: Supplementary file 1 [file NIHMS2104550-supplement-Supplemental_file.docx]

**Supplementary Online Content**

**Appendix Figure 1.** Flowchart of Medicare Current Beneficiary Survey (MCBS) Summary Participant Sampling

**Appendix Table 1.** Comparison of characteristics by Medicare group after weighting (unweighted frequencies and weighted percentages, propensity score and survey weighted)

**Appendix Table 2.** Comparison of characteristics by race/ethnicity and Medicare group (unweighted frequencies and propensity score and survey weighted percentages)

**Appendix Table 3.** Comparison of Medication Adherence between Medicare Beneficiaries in Traditional Medicare and Medicare Advantage, by Race and Ethnicity

**Appendix Table 4.** Preventive and Chronic Disease Care for Medicare Beneficiaries in Traditional Medicare and Medicare Advantage by Dual Enrollment Status

Appendix Table 5. Comparison of Preventive and Chronic Disease Care between Medicare Beneficiaries in Traditional Medicare and Medicare Advantage, by Race and Ethnicity, Restricted to Participants Reporting Stroke or Myocardial Infarction

**Appendix Methods.**

**References** 

**Appendix Figure 1. Flowchart of Medicare Current Beneficiary Survey (MCBS) Summary Participant Sampling** 

**Appendix Table 1. Comparison of characteristics by Medicare group after weighting (unweighted frequencies and weighted percentages, propensity score and survey weighted)**

|  | **No. (%)**^*****^ |  |
| --- | --- | --- |
| **Variable** | **Traditional Medicare** **(n = 44,106** **person-years)** | **Medicare Advantage** **(n = 27,948** **person-years)** |
| **Race/ethnicity** |  |  |
| Non-Hispanic Black | 3,924 (13.8) | 3,391 (13.6) |
| Hispanic | 3,249 (14.8) | 4,182 (13.5) |
| Non-Hispanic White | 34,183 (71.5) | 27,757 (72.8) |
| **Age, mean (SD), years** | 72.2 (12.60) | 72.4 (10.03) |
| **Sex** |  |  |
| Female | 23,351 (55.7) | 15,637 (56.3) |
| Male | 20,755 (44.3) | 12,311 (43.7) |
| **Medicare eligibility reason** |  |  |
| Age | 36,863 (87.6) | 24,338 (86.9) |
| Disability | 6,669 (12.0) | 3,480 (12.7) |
| End stage renal disease | 574 (0.4) | 130 (0.4) |
| **Education** |  |  |
| Less than high school | 7,140 (18.9) | 6,181 (19.1) |
| High school | 12,238 (27.4) | 7,931 (27.6) |
| More than high school | 24,544 (53.7) | 13,722 (53.3) |
| **Marital status** |  |  |
| Married | 21,672 (52.1) | 13,254 (51.0) |
| Widowed | 11,540 (20.8) | 7,569 (22.1) |
| Divorced or separated | 6,448 (21.0) | 4,814 (19.6) |
| Never married | 4,418 (7.3) | 2,293 (7.3) |
| **Area of residence** |  |  |
| Metropolitan | 31,292 (87.1) | 23,287 (86.4) |
| Micropolitan | 8,358 (7.7) | 2,787 (8.1) |
| Rural | 4,450 (5.2) | 1,872 (5.5) |
| **ADL and IADL limitations,**^**†**^ **mean (SD)** | 1.46 (4.20) | 1.56 (3.34) |
| **History of chronic illness** |  |  |
| Diabetes | 15,746 (37.2) | 10,978 (40.3) |
| Hyperlipidemia | 31,572 (73.3) | 20,571 (73.7) |
| Hypertension | 32,615 (74.0) | 21,041 (74.4) |
| Hardening of arteries | 4,295 (8.8) | 2,506 (8.8) |
| Heart attack | 5,656 (11.3) | 3,675 (12.8) |
| Angina | 4,859 (10.0) | 2,957 (10.5) |
| Congestive heart failure | 3,984 (7.5) | 2,354 (7.9) |
| Stroke | 5,641 (11.1) | 3,548 (11.9) |
| Other cardiovascular disease | 14,281 (27.8) | 7,785 (26.8) |
| Skin cancer | 10,644 (20.4) | 5,682 (18.8) |
| Other cancer | 8,999 (19.0) | 5,470 (18.7) |
| Rheumatoid arthritis | 9,251 (21.1) | 6,291 (21.5) |
| Osteoporosis | 8,130 (17.9) | 5,261 (18.3) |
| Alzheimer’s disease | 1,078 (1.9) | 748 (2.0) |
| Other dementia | 1,449 (2.7) | 1,084 (3.1) |
| Depression | 12,307 (27.0) | 7,789 (28.4) |
| Tobacco use (ever) | 23,139 (53.0) | 14,305 (53.0) |
| Vision loss | 3,736 (9.2) | 2,461 (9.2) |
| Hearing loss | 7,795 (17.1) | 4,952 (17.6) |
| **Number of chronic illnesses, mean (SD)** | 3.93 (2.10) | 3.99 (1.67) |

Abbreviations: ADL, activities of daily living; IADL, instrumental activities of daily living; MCBS, Medicare Current Beneficiary Survey.

^*^Percentages are propensity score weighted.

^†^The ADL and IADL limitations score was calculated by summing indicators for the following activities of daily living: difficulty bathing/showering, dressing, eating, getting in or out of bed/chair, walking, using the toilet, using the telephone, doing light housework, doing heavy housework, preparing meals, shopping, and managing money.

**Appendix Table 2. Comparison of characteristics by race/ethnicity and Medicare group (unweighted frequencies and propensity score and survey weighted percentages)**^*****^

| **Variable** | **Non-Hispanic White** |  | **Non-Hispanic Black**^**†**^ |  | **Hispanic**^**†**^ |  |
| --- | --- | --- | --- | --- | --- | --- |
|  | **Traditional Medicare** **(n = 34,183)** **No. (%)** | **Medicare Advantage** **(n = 18,772) No. (%)** | **Traditional Medicare** **(n = 3,924) No. (%)** | **Medicare Advantage** **(n = 3,391) No. (%)** | **Traditional Medicare** **(n = 3,249) No. (%)** | **Medicare Advantage** **(n = 4,182) No. (%)** |
| **Age, mean (SD)** | 72.8 (11.09) | 73.0 (9.59) | 70.4 (11.90) | 70.5 (10.48) | 71.0 (12.54) | 71.3 (10.99) |
| **Sex** |  |  |  |  |  |  |
| Female | 18,001 (54.2) | 10,240 (54.9) | 2,170 (61.9) | 2,181 (63.3) | 1,789 (57.0) | 2,370 (57.7) |
| Male | 16,182 (45.8) | 8,532 (45.1) | 1,754 (38.1) | 1,210 (36.7) | 1,460 (43.0) | 1,812 (42.3) |
| **Medicare eligibility reason** |  |  |  |  |  |  |
| Age | 29,660 (89.7) | 16,765 (89.0) | 2,533 (79.9) | 2,625 (79.1) | 2,490 (84.8) | 3,598 (84.1) |
| Disability | 4,293 (10.1) | 1,955 (10.7) | 1,207 (19.0) | 731 (20.0) | 638 (14.7) | 551 (15.3) |
| End stage renal disease | 230 (0.3) | 52 (0.3) | 184 (1.1) | 35 (0.9) | 121 (0.5) | 33 (0.7) |
| **Education** |  |  |  |  |  |  |
| Less than high school | 3,886 (10.5) | 2,435 (11.3) | 1,135 (29.4) | 1,150 (29.3) | 1,529 (49.7) | 2,264 (51.2) |
| High school | 9,765 (28.8) | 5,771 (29.1) | 1,184 (29.6) | 975 (29.2) | 683 (18.6) | 783 (19.3) |
| More than high school | 20,454 (60.7) | 10,511 (59.6) | 1,572 (40.9) | 1,240 (41.4) | 1,016 (31.7) | 1,124 (29.6) |
| **Marital status** |  |  |  |  |  |  |
| Married | 17,760 (56.1) | 9,580 (54.3) | 1,233 (34.5) | 1,033 (34.4) | 1,378 (49.0) | 1,840 (47.7) |
| Widowed | 9,036 (20.6) | 5,028 (22.0) | 1,003 (23.6) | 992 (24.1) | 818 (20.6) | 1,147 (22.1) |
| Divorced or separated | 4,661 (17.6) | 2,923 (17.8) | 775 (27.2) | 818 (27.7) | 581 (21.9) | 786 (21.5) |
| Never married | 2,708 (5.7) | 1,230 (6.0) | 912 (14.7) | 547 (13.8) | 469 (8.5) | 404 (8.7) |
| **Area of residence** |  |  |  |  |  |  |
| Metropolitan | 23,400 (84.3) | 15,009 (83.9) | 3,064 (89.4) | 2,881 (88.3) | 2,852 (98.5) | 4,101 (98.1) |
| Micropolitan | 6,906 (9.1) | 2,130 (9.1) | 505 (7.7) | 373 (8.4) | 363 (1.0) | 61 (1.3) |
| Rural | 3,871 (6.6) | 1,631 (6.9) | 355 (2.9) | 137 (3.3) | 34 (0.5) | 20 (0.6) |
| **Difficulty performing activities of daily living score, mean (SD)** | 1.28 (1.85) | 1.33 (2.74) | 1.86 (3.76) | 2.00 (2.91) | 1.96 (3.99) | 2.17 (3.88) |
| **History of chronic illness** |  |  |  |  |  |  |
| Diabetes | 11,250 (33.0) | 6,395 (35.0) | 1,728 (46.4) | 1,636 (50.9) | 1,536 (49.2) | 2,150 (52.6) |
| Hyperlipidemia | 24,679 (73.3) | 13,753 (73.4) | 2,584 (69.7) | 2,463 (73.7) | 2,376 (76.8) | 3,216 (76.8) |
| Hypertension  Hardening of arteries | 24,773 (71.2) 3,434 (9.2) | 13,619 (71.5) 1,743 (9.1) | 3,344 (87.6) 238 (6.0) | 2,967 (88.1) 193 (5.4) | 2,438 (74.9) 319 (9.7) | 3,239 (75.5) 406 (9.6) |
| Heart attack | 4,461 (11.5) | 2,505 (13.0) | 431 (10.8) | 385 (11.8) | 378 (11.1) | 571 (13.0) |
| Angina | 3,886 (10.3) | 2,134 (11.2) | 361 (9.9) | 264 (8.7) | 271 (8.2) | 397 (8.7) |
| Congestive heart failure | 3,024 (7.3) | 1,596 (7.7) | 461 (10.4) | 379 (11.9) | 223 (5.5) | 261 (5.7) |
| Stroke | 4,326 (10.9) | 2,312 (11.4) | 590 (12.8) | 541 (15.5) | 375 (10.1) | 450 (10.8) |
| Other cardiovascular disease | 11,728 (30.5) | 5,776 (29.2) | 978 (22.0) | 686 (20.2) | 730 (20.1) | 835 (20.3) |
| Skin cancer | 9,999 (26.8) | 5,236 (25.7) | 63 (1.2) | 19 (0.5) | 247 (7.3) | 240 (5.1) |
| Other cancer | 7,427 (20.3) | 4,055 (20.4) | 588 (16.6) | 502 (14.7) | 494 (14.7) | 614 (14.5) |
| Rheumatoid arthritis | 6,543 (18.3) | 3,632 (18.4) | 1,155 (29.6) | 1,028 (30.1) | 879 (27.0) | 1,213 (28.1) |
| Osteoporosis | 6,526 (18.2) | 3,611 (18.9) | 411 (11.9) | 425 (12.8) | 711 (21.9) | 954 (21.9) |
| Alzheimer’s disease | 735 (1.5) | 433 (1.8) | 129 (2.6) | 102 (2.3) | 121 (2.9) | 167 (2.6) |
| Other dementia | 1,062 (2.4) | 669 (2.9) | 117 (2.4) | 133 (2.9) | 169 (4.3) | 201 (4.1) |
| Depression | 9,258 (26.5) | 5,076 (28.2) | 1,069 (24.2) | 808 (23.5) | 1,140 (32.1) | 1,461 (35.3) |
| Tobacco use (ever) | 18,529 (55.8) | 10,193 (55.9) | 1,799 (52.7) | 1,648 (51.7) | 1,406 (39.6) | 1,681 (41.6) |
| Vision loss | 2,516 (7.3) | 1,373 (7.5) | 489 (12.3) | 407 (12.5) | 462 (15.2) | 523 (14.1) |
| Hearing loss | 6,402 (18.7) | 3,623 (19.2) | 373 (11.2) | 355 (10.6) | 511 (15.0) | 684 (15.9) |
| **Number of chronic illnesses, mean (SD)** | 3.93 (1.85) | 3.99 (2.74) | 3.83 (3.13) | 3.91 (2.33) | 4.01 (2.85) | 4.08 (2.59) |

^*^Percentages are propensity score weighted.

^†^Black individuals were identified based on self-reported race and Hispanic individuals based on self-reported ethnicity; individuals who identified as both Black and Hispanic were included in both sets of analyses.

**Appendix Table 3. Comparison of Medication Adherence between Medicare Beneficiaries in Traditional Medicare and Medicare Advantage, by Race and Ethnicity**^**a**^ 

| **Measures** | **Black** **(n=4,554)**^**b**^ |  | **Non-Hispanic White** **(n=33,262)** |  | **Absolute difference,** **Black–Non-Hispanic White** |  | **Difference in disparities,** **MA vs. TM,** **p.p. (95% CI)** |
| --- | --- | --- | --- | --- | --- | --- | --- |
|  | **MA %** | **TM %** | **MA %** | **TM %** | **MA, p.p. (95% CI)** | **TM, p.p. (95% CI)** |  |
| *Antihypertensives* |  |  |  |  |  |  |  |
| ACE inhibitors^c^ | 81.1 | 77.8 | 85.1 | 84.3 | **-4.0 (-6.5,-1.5)** | **-6.5 (-9.9,-3.1)** | 2.6 (-1.6,6.7) |
| ARBs^d^ | 81.9 | 81.0 | 88.3 | 87.2 | **-6.4 (-9.1,-3.8)** | **-6.2 (-9.8,-2.6)** | -0.2 (-4.7,4.2) |
| CCBs (peripheral)^d^ | 81.6 | 80.6 | 84.1 | 85.0 | **-3.1 (-5.3,-0.8)** | **-4.4 (-7.9,-0.9)** | 1.3 (-2.8,5.5) |
| CCBs (central)^f^ | 87.0 | 80.7 | 83.7 | 82.8 | 3.3 (-1.8,8.4) | -2.1 (-11.1,6.9) | 5.4 (-5.0,15.8) |
| Diuretics^g^ | 76.6 | 73.8 | 81.7 | 80.7 | **-5.1 (-7.8,-2.5)** | **-6.8 (-10.1,-3.6)** | 1.7 (-2.4,5.8) |
| Beta blockers^h^ | 77.1 | 79.5 | 85.1 | 85.2 | **-7.9 (-11.1,-4.8)** | **-5.7 (-8.7,-2.7)** | -2.2 (-6.5,2.0) |
| Alpha/beta blockers^i^ | 76.5 | 75.5 | 83.5 | 81.5 | **-7.0 (-11.7,-2.4)** | **-6.0 (-12.1,0.0)** | -1.0 (-8.7,6.7) |
| *Hypoglycemics* |  |  |  |  |  |  |  |
| Metformin^j^ | 78.7 | 77.0 | 84.5 | 83.2 | **-5.8 (-9.0,-2.6)** | **-6.3 (-10.7,-1.8)** | 0.5 (-5.0,6.0) |
| Sulfonylureas^k^ | 78.3 | 76.0 | 82.8 | 83.6 | -4.5 (-9.8,0.8) | **-7.6 (-15.1,-0.2)** | 3.2 (-6.0,12.3) |
| DPP IV inhibitors^l^ | 81.0 | 77.5 | 77.7 | 75.9 | 3.4 (-4.1,10.8) | 1.6 (-4.1,10.8) | 1.8 (-9.0,12.5) |
| Thiazolidinediones^m^ | 74.1 | 66.8 | 80.6 | 84.0 | -6.5 (-17.3,4.3) | **-17.3 (-26.8,-7.8)** | 10.8 (-3.6,25.2) |
| SGLT-2 inhibitors^n^ | 75.3 | 68.8 | 73.8 | 72.9 | 1.5 (-10.9,13.8) | -4.1 (-20.4,12.1) | 5.6 (-14.8,26.0 |
| *Lipid lowering drugs* |  |  |  |  |  |  |  |
| Statins^o^ | 82.1 | 77.6 | 86.2 | 86.5 | **-4.1 (-6.0,-2.2)** | **-8.9 (-11.6,-6.2)** | **4.8 (1.5,8.1)** |
| **Measures** | **Hispanic** **(n=4,527)** |  | **Non-Hispanic White** **(n=33,262)** |  | **Absolute difference,** **Hispanic–Non-Hispanic White** |  | **Difference in disparities,** **MA vs. TM,** **p.p. (95% CI)** |
|  | **MA %** | **TM %** | **MA %** | **TM %** | **MA, p.p. (95% CI)** | **TM, p.p. (95% CI)** |  |
| *Antihypertensives* |  |  |  |  |  |  |  |
| ACE inhibitors^c^ | 80.9 | 82.3 | 85.1 | 84.3 | **-4.1 (-7.1,-1.2)** | -2.0 (-5.6,1.7) | -2.2 (-6.9,2.5) |
| ARBs^d^ | 84.7 | 80.7 | 88.3 | 87.2 | **-3.6 (-5.7,-1.5)** | **-6.6 (-13.0,-0.2)** | 3.0 (-3.7,9.7) |
| CCBs (peripheral)^e^ | 80.3 | 79.1 | 84.1 | 85.0 | **-3.8 (-7.0,-0.6)** | **-5.9 (-9.6,-2.2)** | 2.1 (-2.8,7.0) |
| CCBs (central)^f^ | 83.1 | 81.0 | 83.7 | 82.8 | -0.6 (-7.9,6.8) | -1.9 (-9.5,5.7) | 1.3 (-9.4,12.0) |
| Diuretics^g^ | 81.4 | 77.9 | 81.7 | 80.7 | -0.3 (-2.9,2.2) | -2.8 (-6.3,0.8) | 2.4 (-1.8,6.7) |
| Beta blockers^h^ | 83.7 | 85.1 | 85.1 | 85.2 | -1.3 (-3.5,0.9) | -0.0 (-3.2,3.1) | -1.3 (-5.1,2.5) |
| Alpha/beta blockers^i^ | 79.1 | 84.3 | 83.5 | 81.5 | **-4.4 (-8.7,-0.1)** | 2.8 (-4.3,10.0) | -7.3 (-15.4,0.8) |
| *Hypoglycemics* |  |  |  |  |  |  |  |
| Metformin^j^ | 80.4 | 79.6 | 84.5 | 83.2 | **-4.1 (-6.7,-1.5)** | -3.7 (-8.2,0.9) | -0.4 (-5.7,4.7) |
| Sulfonylureas^k^ | 75.8 | 75.6 | 82.8 | 83.6 | **-7.0 (-11.9,-2.1)** | -7.9 (-17.5,1.6) | .09 (-9.8,11.7) |
| DPP IV inhibitors^l^ | 79.4 | 83.0 | 77.7 | 75.9 | 1.7 (-4.1,7.6) | **7.1 (0.4,13.9)** | -5.4 (-14.3,3.5) |
| Thiazolidinediones^m^ | 81.6 | 73.8 | 80.6 | 84.0 | 1.0 (-6.6,8.7) | **-10.3 (-18.8,-1.7)** | 11.3 (-0.2,22.8) |
| SGLT-2 inhibitors^n^ | 71.7 | 78.8 | 73.8 | 72.9 | -2.2 (-11.7,7.3) | 5.9 (-9.6,21.4) | -8.0 (-26.2,10.1) |
| *Lipid lowering drugs* |  |  |  |  |  |  |  |
| Statins^o^ | 82.2 | 80.7 | 86.2 | 86.5 | **-4.0 (-5.7,-2.3)** | **-5.8 (-8.9,-2.7)** | 1.8 (-1.7,5.3) |

Abbreviations: TM, Traditional Medicare; MA, Medicare Advantage; p.p., percentage points; ACE, angiotensin-converting enzyme; ARB, angiotensin II receptor blocker; CCB, calcium channel blocker; DPP, dipeptidyl peptidase; SGLT, sodium glucose cotransporter.

^a^Results are propensity score weighted.

^b^n values reported are in person-years.

^c^ACE inhibitors include the following medications: amlodipine/benazepril (Lotrel), amlodipine/benazepril, benazepril/hydrochlorothiazide (HCTZ), benazepril, captopril, captopril/HCTZ, enalapril/HCTZ, enalapril/HCTZ, enalapril, fosinopril, fosinopril/HCTZ, lisinopril (Prinivil), lisinopril/HCTZ, moexipril/HCTZ, moexipril, perindopril, quinapril/HCTZ, quinapril (Accupril), ramipril (Altace), trandolapril, trandolapril/verapamil ER.

^d^ARBs include the following medications: amlodipine/olmesartan (Azor), amlodipine/olmesartan/HCTZ (Tribenzor), amlodipine/valsartan (Exforge), amlodipine/valsartan/HCTZ (Exforge HCT), amlodipine/valsartan, azilsartan (Edarbi), candesartan (Atacand), candesartan/HCTZ, azilsartan/chlorthalidone (Edarbyclor), irbesartan (Avapro), irbesartan/HCTZ, losartan (Cozaar), losartan/HCTZ, olmesartan/amlodipine/HCTZ, olmesartan (Benicar), olmesartan/HCTZ (Benicar HCT), telmisartan (Micardis), telmisartan/amlodipine, telmisartan/HCTZ (Micardis HCT), valsartan (Diovan), valsartan/HCTZ (Diovan HCT).

^e^CCBs (peripheral) include the following medications: amlodipine (Norvasc), amlodipine/benazepril (Lotrel), amlodipine/valsartan, amlodipine/olmesartan (Azor), amlodipine/olmesartan/HCTZ (Tribenzor), amlodipine/valsartan (Exforge), amlodipine/valsartan/HCTZ (Exforge HCT), felodipine ER, isradipine, nifedipine (Afeditab CR, Nifedical XL), nifedipine ER osmotic release (Procardia XL), nimodipine, nisoldipine ER, olmesartan/amlodipine/HCTZ, telmisartan amlodipine.

^f^CCBs (central) include the following medications: diltiazem HCl (Cardizem CD, Cardizem LA, Cartia XT, Dilt XR, Matzim LA, Taztia XT), Verapamil HCl (Calan SR).

^g^Diuretics include the following medications: aliskiren/HCTZ (Tekturna HCT), amlodipine/olmesartan/HCTZ (Tribenzor), amlodipine/valsartan (Exforge), amlodipine/valsartan/HCTZ (Exforge HCT), azilsartan/chlorthalidone (Edarbyclor), bumetanide, candesartan/HCTZ, chlorothiazide (Diuril), chlorthalidone, ethacrynic acid (Edecrin), furosemide (Lasix), hydrochlorothiazide (HCTZ), indapamide, irbesartan/HCTZ, losartan/HCTZ, methyclothiazide, methyldopa/HCTZ, metolazone, olmesartan/amlodipine/HCTZ, olmesartan/medoxomil/HCTZ (Benicar HCT), telmisartan/HCTZ (Micardis HCT), torsemide, valsartan/HCTZ (Diovan HCT).

^h^Beta blockers include the following medications: acebutolol, atenolol, atenolol/chlorthalidone, bisoprolol/HCTZ, bisoprolol, metoprolol/HCTZ (Dutoprol), metoprolol succinate ER (Kapspargo Sprinkle, Metoprolol Tartrate, Toprol XL), nadolol, nadolol/bendroflumethiazide, nebivolol (Bystolic), pindolol, propranolol/HCTZ, propranolol, propranolol ER, sotalol (Sotalol AF).

^i^Alpha/beta blockers include the following medications: carvedilol, carvedilol ER (Coreg CR), labetalol, methyldopa/HCTZ.

^j^Metformin includes the following medications: metformin (Fortamet, Glumetza, Glucophage, Riomet), metformin/alogliptin, metformin/canagliflozin (Invokamet XR), metformin/dapagliflozin (Xigduo XR), metformin/empagliflozin (Synjardy, Synjardy XR), metformin/glipizide, metformin/glyburide, saxagliptin/metformin (Kombiglyze XR), metformin/linagliptin (Jentadueto, Jentadueto XR), metformin/pioglitazone, metformin/sitagliptin (Janumet, Janumet XR).

^k^Sulfonylureas include the following medications: glimepiride, glipizide (Glucotrol XL), glipizide/metformin, glipizide ER, glipizide XL, glyburide, glyburide/metformin, glyburide micronized, pioglitazone/glimepiride.

^l^DPP IV inhibitors include the following medications: alogliptin/metformin, alogliptin/pioglitazone (Oseni), alogliptin/benzoate (Nesina), empagliflozin/linagliptin (Glyxambi), linagliptin (Tradjenta), linagliptin/metformin (Jentadueto), saxagliptin (Onglyza), saxagliptin/metformin (Kombiglyze XR), sitagliptin (Januvia), sitagliptin/metformin (Janumet, Janumet XR).

^m^Thiazolidinediones include the following medications: pioglitazone, alogliptin/pioglitazone (Oseni), pioglitazone/metformin, pioglitazone/glimepiride.

^n^SGLT 2 inhibitors include the following medications: canagliflozin (Invokana), canagliflozin/metformin (Invokamet, Invokamet XR), dapagliflozin (Farxiga), dapagliflozin/metformin (Xigduo XR), empagliflozin (Jardiance), empagliflozin/linagliptin (Glyxambi), empagliflozin/metformin (Synjardy, Synjardy XR).

^o^Statins include the following medications: atorvastatin (Lipitor), fluvastatin (Lescol XL), pitavastatin (Livalo), lovastatin, niacin/lovastatin (Advicor), niacin/simvastatin (Simcor), pravastatin, rosuvastatin (Crestor), simvastatin (Zocor).

**Appendix Table 4. Preventive and Chronic Disease Care for Medicare Beneficiaries in Traditional Medicare and Medicare Advantage by Dual Enrollment Status**^*****^

| **Measures** | **Race/** **Ethnicity**^**†**^ | **Non-dual** |  |  |  | **Dual** |  |  |  |
| --- | --- | --- | --- | --- | --- | --- | --- | --- | --- |
|  |  | MA | TM | MA-TM difference (95% CI) | Difference in disparity vs. White (95% CI) | MA | TM | MA-TM difference (95% CI) | Difference in disparity vs. White (95% CI) |
| Preventive care index (0-5), mean | White | 3.94 | 3.86 | 0.08 (0.05, 0.11) | -- | 3.59 | 3.38 | 0.21 (0.12, 0.29) | -- |
|  | Black | 3.74 | 3.57 | 0.17 (0.07, 0.28) | 0.10 (-0.02, 0.21) | 3.58 | 3.27 | 0.31 (0.18, 0.45) | 0.11 (-0.05, 0.27) |
|  | Hispanic | 3.76 | 3.71 | 0.05 (-0.07, 0.17) | -0.03 (-0.15, 0.09) | 3.59 | 3.41 | 0.18 (0.07, 0.30) | -0.02 (-0.17, 0.12) |

^*^Results are propensity score weighted.

^†^Black individuals were identified based on self-reported race and Hispanic individuals based on self-reported ethnicity; individuals who identified as both Black and Hispanic were included in both sets of analyses.

**Appendix Table 5**. **Comparison of Preventive and Chronic Disease Care between Medicare Beneficiaries in Traditional Medicare and Medicare Advantage, by Race and Ethnicity, Restricted to Participants Reporting Stroke or Myocardial Infarction***

| **Measures** | **Black**^†^ **(n=1,711)**^‡^ |  | **White** **(n=12,200)** |  | **Absolute difference, Black–White**^§^ |  | **Difference in disparities, MA vs. TM, p.p. (95% CI)**^§^ | **Unadjusted p values** |
| --- | --- | --- | --- | --- | --- | --- | --- | --- |
|  | **TM, %** | **MA, %** | **TM, %** | **MA, %** | **TM, p.p.** **(95% CI)** | **MA, p.p.** **(95% CI)** |  |  |
| *Preventive Care* |  |  |  |  |  |  |  |  |
| Annual wellness visit^‖^ | 22.1 | 43.9 | 44.7 | 54.1 | **-22.5 (-32.9, -12.2)** | **-10.2 (-20.3, -0.2)** | 12.3 (-2.1, 26.7) | 0.09 |
| Blood pressure check | 91.8 | 94.9 | 92.0 | 92.0 | -0.2 (-3.6, 3.3) | **2.9 (0.7, 5.1)** | 3.1 (-1.0, 7.1) | 0.14 |
| Cholesterol test | 92.6 | 96.0 | 93.3 | 93.7 | -0.7 (-4.1, 2.6) | **2.3 (0.3, 4.3)** | 3.0 (-1.0, 7.0) | 0.14 |
| Influenza vaccine | 61.4 | 65.4 | 75.4 | 78.1 | **-14.0 (-20.9, -7.0)** | **-12.7 (-18.9, -6.6)** | 1.3 (-7.8, 10.3) | 0.79 |
| Pneumococcal vaccine | 75.0 | 75.2 | 86.0 | 86.7 | **-11.0 (-18.4, -3.5)** | **-11.5 (-17.1, -5.9)** | -0.5 (-9.4, 8.3) | 0.91 |
| Colorectal cancer screening | 61.7 | 67.1 | 66.8 | 69.7 | -5.1 (-10.7, 0.5) | -2.6 (-6.9, 1.7) | 2.5 (-4.7, 9.7) | 0.50 |
| Breast cancer screening | 41.5 | 44.4 | 35.4 | 33.9 | 6.1 (-3.1, 15.3) | 10.5 (3.7, 17.3) | 4.4 (-6.8, 15.7) | 0.44 |
| Preventive care index^¶^ (0-5), mean | 3.41 | 3.69 | 3.80 | 3.91 | **-0.39 (-0.53, -0.25)** | **-0.21 (-0.34, -0.09)** | 0.18 (-0.01, 0.37) | 0.07 |
| *Diabetes Management*^#^ |  |  |  |  |  |  |  |  |
| Hemoglobin A1C (≥2 in past year) | 69.2 | 73.2 | 66.6 | 71.3 | 2.6 (-6.0, 11.2) | 1.9 (-6.4, 10.1) | -0.7 (-12.4, 11.0) | 0.91 |
| Eye exam | 44.9 | 60.5 | 55.5 | 54.4 | **-10.7 (-16.1, -5.2)** | **6.1 (1.0, 11.2)** | **16.8 (9.5, 24.0)** | **<0.001** |
| **Measures** | **Hispanic**^†^ **(n=1,573)**^‡^ |  | **White** **(n=12,200)** |  | **Absolute difference, Hispanic–White**^§^ |  | **Difference in disparities, MA vs. TM, p.p. (95% CI)**^§^ |  |
|  | **TM, %** | **MA, %** | **TM, %** | **MA, %** | **TM, p.p.** **(95% CI)** | **MA, p.p.** **(95% CI)** |  | **Unadjusted p values** |
| *Preventive Care* |  |  |  |  |  |  |  |  |
| Annual wellness visit^‖^ | 39.5 | 45.8 | 44.7 | 54.1 | -5.1 (-18.8, 8.5) | -8.3 (-19.0, 2.3) | -3.2(-20.6, 14.1) | 0.72 |
| Blood pressure check | 94.3 | 93.3 | 92.0 | 91.9 | 2.3 (-0.2, 4.9) | 1.4 (-1.1, 3.8) | -1.0 (-4.4, 2.5) | 0.58 |
| Cholesterol test | 94.7 | 93.8 | 93.3 | 93.7 | 1.3 (-1.4, 4.1) | 0.1 (-2.4, 2.6) | -1.2 (-4.8, 2.3) | 0.50 |
| Influenza vaccine | 73.6 | 62.8 | 75.4 | 78.1 | -1.8 (-9.0, 5.3) | **-15.3 (-21.9, -8.8)** | **-13.5 (-22.9, -4.1)** | **0.01** |
| Pneumococcal vaccine | 81.1 | 71.5 | 86.0 | 86.7 | -4.9 (-11.4, 1.6) | **-15.2 (-21.0, -9.3)** | **-10.2 (-18.6, -1.9)** | **0.02** |
| Colorectal cancer screening | 60.3 | 67.4 | 66.8 | 69.7 | **-6.6 (-12.0, -1.1)** | -2.4 (-6.5, 1.7) | 4.2 (-2.4, 10.8) | 0.21 |
| Breast cancer screening | 38.1 | 43.1 | 35.4 | 33.9 | 2.7 (-5.5, 10.8) | **9.2 (2.4, 16.1)** | 6.6 (-3.9, 17.0) | 0.22 |
| Preventive care index^¶^ (0-5), mean | 3.70 | 3.63 | 3.80 | 3.91 | -0.10 (-0.24, 0.03) | **-0.28 (-0.41, -0.14)** | -0.17 (-0.36, 0.01) | 0.06 |
| *Diabetes Management*^#^ |  |  |  |  |  |  |  |  |
| Hemoglobin A1C (≥2 in past year) | 56.4 | 60.2 | 66.6 | 71.3 | -10.2 (-23.9, 3.5) | **-11.1 (-19.9, -2.4)** | -0.9 (-17.3, 15.4) | 0.91 |
| Eye exam | 56.6 | 57.2 | 55.5 | 54.4 | 1.0 (-5.2, 7.2) | 2.8 (-2.7, 8.3) | 1.8 (-6.5, 10.1) | 0.67 |

Abbreviations: TM, Traditional Medicare; MA, Medicare Advantage; p.p., percentage points.

^*^Results are propensity score weighted.

^†^Black and Hispanic individuals were identified based on self-reported race; individuals who identified as both Black and Hispanic were included in both sets of analyses.

^‡^n values reported are in person-years.

^§^Bolded numbers indicates significance at P<0.05.

^‖^Annual wellness visit data were collected only in 2020.

^¶^Preventive care index is a sum of indicators for blood pressure check, cholesterol test, influenza vaccine, pneumococcal vaccine, and colorectal cancer screening (range 0-5, depending on number of “yes” responses).

^#^Sample is restricted to only participants who reported a diabetes diagnosis.

**Appendix Methods**

*Detailed MCBS study design.* MCBS participants are selected using a three-stage cluster sampling design. The primary sampling unit is a defined geographic area, which can be a metropolitan area or a combination of rural counties. The secondary sampling unit is composed of individual or combined census tracts. Finally, the tertiary sampling unit is individual beneficiaries, who are selected via systematic random sampling with random starts. Data collection was traditionally done in person. Computer-assisted data collection was used in the 2010s. However, disruptions to in-person activities during the COVID-19 pandemic caused data collection to shift to phone-only for a part of 2020. We used the main survey sampling weights in all analyses except the medication adherence analysis, where cost-supplement-specific weights were used. The MCBS derived variable D_MADV was used to categorize individuals as having Medicare Advantage (MA) vs. traditional Medicare (TM).

*Difference-in-disparities analyses.* We used weighted logistic regression models to produce marginal estimates of outcomes in each Medicare type-race and ethnicity subgroup. Contrasts were used to estimate racial and ethnic disparities within TM and MA separately, and then to estimate differences in disparities between the two Medicare types. We reported marginal outcome estimates as weighted percentages for categorical variables and weighted means for continuous variables. 

*Medication adherence analyses.* For our secondary analysis of medication adherence, we repeated the procedure from our primary analysis using medication possession ratios (MPRs, capped at 90 days)^1,2^ for each medication as our outcome (eTable 3): antihypertensive medications [angiotensin-converting-enzyme (ACE) inhibitors, angiotensin II receptor blockers (ARBs), peripheral calcium channel blockers (CCBs), central CCBs, diuretics, beta blockers, alpha/beta blockers], diabetes medications [metformin, sulfonylureas, dipeptidyl peptidase (DPP) IV inhibitors, thiazolidinediones, sodium-glucose co-transporter-2 (SGLT-2) inhibitors], and statins.

*Supplemental insurance analyses.* For our secondary analysis of supplemental Medicaid insurance, we split the sample by supplemental Medicaid enrollment (dual enrolled vs. not dual enrolled). We repeated the procedure from our primary analysis, using logistic regression to estimate disparities within each Medicare type, stratified by dual enrollment status (eTable 4).

**References**

1.	Vink NM, Klungel OH, Stolk RP, Denig P. Comparison of various measures for assessing medication refill adherence using prescription data. *Pharmacoepidemiol Drug Saf*. 2009;18(2):159-65. doi:10.1002/pds.1698

2.	Cramer JA, Benedict A, Muszbek N, Keskinaslan A, Khan ZM. The significance of compliance and persistence in the treatment of diabetes, hypertension and dyslipidaemia: a review. *Int J Clin Pract*. 2008;62(1):76-87. doi:10.1111/j.1742-1241.2007.01630.x
